# Predictors of hepatitis B vaccination status in healthcare workers in Belgrade, Serbia, December 2015

**DOI:** 10.2807/1560-7917.ES.2017.22.16.30515

**Published:** 2017-04-20

**Authors:** Darija Kisic-Tepavcevic, Milena Kanazir, Tatjana Gazibara, Gorica Maric, Natasa Makismovic, Goranka Loncarevic, Tatjana Pekmezovic

**Affiliations:** 1 Institute of Epidemiology, Faculty of Medicine, University of Belgrade, Belgrade, Serbia; 2 These authors contributed equally to this article; 3 Institute of Public Health of Serbia ‘Dr Milan Jovanovic Batut’, Belgrade, Serbia

**Keywords:** hepatitis B, vaccination, health-care workers, knowledge, acceptance

## Abstract

Despite the availability of a safe and effective vaccine since 1982, overall coverage of hepatitis B vaccination among healthcare workers (HCWs) has not reached a satisfactory level in many countries worldwide. The aim of this study was to estimate the prevalence of hepatitis B vaccination, and to assess the predictors of hepatitis B vaccination status among HCWs in Serbia. Of 380 randomly selected HCWs, 352 (92.6%) were included in the study. The prevalence of hepatitis B vaccination acceptance was 66.2%. The exploratory factor analyses using the vaccination-refusal scale showed that items clustered under ‘threat of disease’ explained the highest proportion (30.4%) of variance among those declining vaccination. The factor analyses model of the potential reasons for receiving the hepatitis B vaccine showed that ‘social influence’ had the highest contribution (47.5%) in explaining variance among those vaccinated. In the multivariate adjusted model the following variables were independent predictors of hepatitis B vaccination status: occupation, duration of work experience, exposure to blood in the previous year, and total hepatitis B-related knowledge score. Our results highlight the need for well-planned national policies, possibly including mandatory hepatitis B immunisation, in the Serbian healthcare environment.

## Introduction

Hepatitis B infection is a major cause of occupational disease among healthcare workers (HCWs) worldwide. It has been estimated that every year between 600,000 and 800,000 cut and puncture injures occur in this professional group [1,2]. Furthermore, the global annual proportion of HCWs exposed to hepatitis B virus (HBV) has been estimated at 5.9%, corresponding to ca 66,000 HBV infections [2,3]. In developing countries, 40–60% of HBV infections in HCWs were attributed to professional hazard, while in developed countries the attributed fraction was less than 10% due to greater vaccination coverage [4].

Despite the availability of a safe and effective vaccine since 1982, the overall prevalence of hepatitis B vaccination in this cohort at risk has not reached a satisfactory level [4-7]. Studies have revealed that HCWs’ acceptance of this vaccination ranges from 15% in Africa, to slightly more than 75% in Australia, New Zealand and the United States [8-12]. While ca 90% of the HCWs are aware of the necessity of the hepatitis B vaccination in the workplace, only half of them complete the HBV vaccination course [8,9]. These findings suggest that low rates of hepatitis B vaccination in HCWs, despite the well-recognised high professional risk, are difficult to comprehend and explain. Various potential reasons have been proposed for failure to receive the hepatitis B vaccine, including fear of side effects, availability and cost [13]. However, determinants of acceptance are likely to be multifaceted and have tended to change over time as data regarding effectiveness and safety of this vaccine have accumulated. Nowadays, it is clear that issues surrounding hepatitis B vaccine-related attitudes in HCWs are more complex and comprehensive. There are numerous psychological, occupational and behavioural factors that should be taken into consideration when predicting hepatitis B vaccination acceptance in this at-risk cohort.

In Serbia, there are very few data available on the hepatitis B vaccination status of HCWs, although this vaccine is mandatory for occupationally exposed HCWs [14]. Moreover, the determinants of hepatitis B vaccination uptake among Serbian healthcare providers are not well understood. We therefore aimed to estimate the prevalence of hepatitis B vaccination and assess the predictors of hepatitis B vaccination status among HCWs at a national healthcare centre in Serbia.

## Material and methods

A cross-sectional study design was applied in order to explore predictors of hepatitis B vaccination status among HCWs in the largest clinical centre in Serbia.

### Participants and settings

The Clinical Centre of Serbia, with 41 organisational units (of which 23 are clinics) and 3,500 beds, is Serbia’s national referral hospital, located in the capital city, Belgrade, which has ca 1.6 million inhabitants. It is affiliated with the Faculty of Medicine of the University of Belgrade, the state university with ca 1,200 faculty staff.

The HBV vaccine has been provided free of charge to occupationally exposed employees in Clinical Centre of Serbia since 1989. However, despite legal rules, the vaccine has been offered sporadically (depending on socioeconomic situation and availability of vaccine) at the time of employment and on request, but it is also mandatory after evaluation of high-risk occupational injury. However, organised public health efforts to increase the hepatitis B vaccination compliance throughout the Clinical Centre of Serbia have not yet been realised.

A random sample of HCWs stratified by occupation was selected from the list of employees in December 2015, with the sample structure reflecting occupational distribution within the Clinical Centre of Serbia. The sample comprised 7.1% of the employees at the Clinical Centre of Serbia.

All participants provided signed informed consent. The study was approved by the Ethical Committee of the Faculty of Medicine, University of Belgrade.

The relevant data in this study were collected by questionnaire that was derived and adapted from other surveys [13,15]. After translation into Serbian its validity was assessed by the authors (DKT, MK) using standard methodology (assessment of reliability and factor analysis). The questionnaire consisted of four parts. The first comprised demographic and professional data about sex, age, marital status, occupation, work site and duration of work experience. The second part of the questionnaire consisted of 30 statements (offering yes/no answers), created to explore HCWs’ knowledge levels towards HBV infection, including the nature of the disease and its transmission, symptoms and complications, and possibilities for prevention and treatment (Table 1).

**Table 1:** Percentages of correct hepatitis B knowledge answers, questionnaire completed by healthcare workers at the Clinical Centre of Serbia, December 2015 (n=352)

Statements	Correct answers	
	Number	%
1. Hepatitis B is caused by a virus	334	94.9
2. Hepatitis B can be spread by mosquitoes	274	77.8
3. Hepatitis B can be spread through close personal contact such as talking and kissing	307	87.2
4. Hepatitis B can be spread through sharing injecting equipment, such as needles and operation tools	337	95.7
5. Hepatitis B can be transferred from mother to fetus	307	87.2
6. Hepatitis B is spread through blood-to-blood contact	336	95.5
7. Having a medical and/or dental procedure increases a person’s likelihood of contracting hepatitis B	319	90.6
8. Hepatitis B is spread through the air in an enclosed environment	291	82.7
9. Hepatitis B is commonly spread by sexual transmission	332	94.3
10. Some people with hepatitis B were infected through unsterile tattooing	322	91.5
11. Some people with hepatitis B were infected through blood transfusions	328	93.2
12. Hepatitis B can be spread by sharing dishes with HBV positive patients	256	72.7
13. HBV can spread from one person to another within a family	190	54.0
14. Once you have had hepatitis B, you cannot catch it again because you are immune	209	59.4
15. HBV can be transferred through colonoscopy or endoscopy tools	258	73.3
16. HBV can be transferred through mother’s milk to the infant	311	88.4
17. After entry of HBV to the body, symptoms appear after 1 to 3 days	301	85.5
18. Hepatitis B can lead to cirrhosis	144	40.9
19. An individual can have hepatitis B antibodies without being currently infected with the virus	308	87.5
20. Hepatitis B is associated with an increased risk of liver cancer	251	71.3
21. A person can be infected with HBV and not have any symptoms of the disease	268	76.1
22. Symptoms of hepatitis B infection always appear	263	74.7
23. People with hepatitis B should be restricted from working in the food industry	173	49.1
24. There is a vaccine for hepatitis B	341	96.9
25. Special diet is recommended for patients with hepatitis B	232	65.9
26. Pregnant women should not receive the vaccine against hepatitis B	171	48.6
27. Newborn children should not receive the vaccine against hepatitis B	227	64.5
28. Vaccination against hepatitis B is obligatory for all persons employed in healthcare institutions who come in direct contact with infectious materials	311	88.4
29. There is a pharmaceutical treatment available for hepatitis B	291	82.7
30. The vaccine can be used for the treatment of hepatitis B	250	71.0

HBV: hepatitis B virus.

Each correct answer in this set of items was awarded 1 point. Therefore, the total HBV-related knowledge score represented a range between a minimum of 0 and maximum of 30 points. The third part contained the questions related to hepatitis B vaccination status of respondents, as well as a number of issues related to hazardous contact with blood and blood products in the workplace.

The general estimate of voluntary vaccination acceptance in our sample was assessed using the frequency of participant’s influenza immunisation as an indicator. Furthermore, in order to control for a possible confounding effect of general acceptance of a legally mandated preventive health measure, HCWs were also asked to categorise their frequency of seat belt use when driving the car, which is required by law in the Republic of Serbia.

The last part of the questionnaire consisted of both 13-item vaccination-acceptance and 15-item vaccination-refusal scales. The respondents completed the scale that was relevant to their hepatitis B vaccination status. Items in the scales were designed to explain HCWs’ hepatitis B vaccination status and their potential reasons for compliance or non-compliance. Therefore, workers were asked to assess the relative contribution of each item on a seven-point Likert scale, with response options ranging from ‘not important’ (one point) to ‘very important’ (seven points). The total score in each domain was calculated as the mean Likert point with corresponding standard deviation.

### Statistical analyses

Normality of distribution was tested by using the Kolmogorov-Smirnov test. Data were presented as mean ± standard deviation for continuous variables and as absolute numbers and percentage for discrete variables. Differences between groups were assessed by t-test and chi-squared test. A p value of less than 0.05 was considered as statistically significant.

Internal reliabilities of the vaccination-refusal and vaccination-acceptance scales were assessed using Cronbach’s alpha coefficient for multiple item scales, which ranges from 0 to 1, with 1 representing perfect reliability.

In order to assess the allocation of items into domains (construct validity) of the vaccination-refusal and acceptance scales, exploratory factor analyses (principal component analysis with varimax rotation) were conducted. A factor was considered important if its eigenvalue exceeded 1.0.

Independent predictors of hepatitis B vaccine status among HCWs were identified using a series of logistic regression models based on heterogeneous factors with potential confounding effects. All potential covariates were first analysed in a univariate unadjusted regression model with hepatitis B vaccination status as dependent variable. Subsequently, a multivariate logistic regression analysis was performed to test whether possible predictors remained statistically significant. This adjusted analysis included all covariates that appeared to be associated (p < 0.05) with the outcome following the univariate unadjusted analysis.

## Results

Of 380 randomly selected HCWs, 367 (96.6%) were enrolled in the study, but only 356 (93.7%) provided all relevant information. Of these 356, four potential participants reported a history of hepatitis B and were excluded from all subsequent analyses. Thus, the total sample size in our survey comprised 352 HCWs, which corresponded to a statistical power of 0.843, with 95% confidence interval and probability level of α = 0.05.

Overall, the prevalence of HCW vaccinated against hepatitis B was 66.2%. Additionally, among workers who had been vaccinated, 189 (81.1%) had completed the three-dose course, while 27 (11.6%) had received two doses, and seven (3.0%) one dose. Ten (4.3%) HCWs in our study did not know the number of doses they had received. Comparison of participants’ demographic and professional characteristics by hepatitis B vaccination status is presented in Table 2.

**Table 2:** Comparison of participants’ demographic and professional characteristics by hepatitis B vaccination status, questionnaire completed by healthcare workers at the Clinical Centre of Serbia, December 2015 (n=352)

	Unvaccinated (n = 119)		Vaccinated (n = 233)		p value
**Sex** Male Female	**No.** 23 96	**%** 26.7 36.1	**No.** 63 170	**%** 73.3 63.9	0.118
**Аgе** (years) Mean ± SD	41.5 ± 9.5		37.8 ± 8.8		**< 0.001**
**Marital status** Single (never married) Married/cohabiting Separated/divorced Widowed	**No.** 33 74 11 1	**%** 29.7 35.1 39.3 50.0	**No.** 78 137 17 1	**%** 70.3 64.9 60.7 50.0	0.663
**Occupation** Physicians (specialist) Physicians undergoing specialisation Physicians without specialisation Nurses Medical technologists Laboratory technologists Administrative staff Sanitary workers Others	**No.** 9 7 3 58 11 3 5 11 12	**%** 19.6 17.9 42.9 32.0 32.4 50.0 71.4 78.6 66.7	**No.** 37 32 4 123 23 3 2 3 6	**%** 80.4 82.1 57.1 68.0 67.6 50.0 28.6 21.4 33.3	**< 0.001**
**Work site** Operating theatre Accident and emergency, haemodialysis Specialty ward/Intensive care unit Laboratory Inpatient wards Others	**No.** 24 14 8 2 33 38	**%** 22.9 37.8 22.9 33.3 29.5 66.7	**No.** 81 23 27 4 79 19	**%** 77.1 62.2 77.1 66.7 70.5 33.3	**< 0.001**
**Duration of work experience (years)** Mean ± SD	19.3 ± 10.8		14.1 ± 9.2		**< 0.001**
**Episodes of exposure of unprotected skin/mucous membranes to blood in the past year** 0 1–5 6–10 More than 10	**No.** 32 36 10 41	**%** 55.2 34.0 19.6 29.9	**No.** 26 70 41 96	**%** 44.8 66.0 80.4 70.1	**0.001**
**Episodes of sharps injuries in the past year** 0 1 2 More than 2	**No.** 56 12 19 32	**%** 38.4 20.0 19.6 32.0	**No.** 90 48 27 68	**%** 61.6 80.0 80.4 68.0	0.051

SD: standard deviation.

Employees who had either initiated or completed vaccination were significantly younger (37.8 ± 8.8 years old) than those who were unvaccinated (41.5 ± 9.5 years old). Slightly more men (73.3%) than women (63.9%) reported vaccination against hepatitis B (p = 0.118). Vaccination uptake varied significantly by occupation and work site, with predominantly higher proportions vaccinated among physicians and those working in surgical and intensive care units. Overall, 58.5% (206/352) of workers reported sharps injury, and 73.6% (259/352) reported unprotected blood mucocutaneous exposure in the past year. Statistically significantly higher rates of hepatitis B vaccination were observed in HCW sub-cohorts who had at least one episode of sharps injury and/or exposure of skin/mucous membranes to blood in the past year.

The overall reliabilities of the vaccination-refusal and vaccination-acceptance scales, as estimated by Cronbach’s alpha coefficients, were 0.812 and 0.881, respectively (Table 3).

**Table 3:** The reliability of hepatitis B vaccination-refusal and -acceptance scales, questionnaire completed by healthcare workers at the Clinical Centre of Serbia, December 2015 (n=352)

**Vaccination refusal**	
**Factors**	**Cronbach’s alpha**
Threat of disease	0.872
Knowledge of disease	0.860
Social influence	0.805
Access to care	0.727
Risk denial	/^a^
**Total**	**0.812**
**Vaccination acceptance**	
**Factors**	**Cronbach’s alpha**
Threat of disease	0.726
Knowledge of disease	0.767
Social influence	0.884
**Total**	**0.881**

The loading weights obtained in the exploratory factor analyses of these scales are shown in Tables 4 and 5.

**Table 4:** Exploratory factor analysis of the reasons for not receiving the hepatitis B vaccine, questionnaire completed by healthcare workers at the Clinical Centre of Serbia, December 2015 (n=352)

Reasons	Mean score (n = 109)	Factor 1: Threat of disease	Factor 2: Knowledge of disease	Factor 3: Social influence	Factor 4: Access to care	Factor 5: Risk denial
Concern about possible jaundice due to vaccination	2.9 ± 1.8	**0.848**	0.041	0.128	0.143	0.040
Concern about possible HIV infection due to vaccination	1.8 ± 1.1	**0.672**	0.212	0.127	0.320	-0.307
Concern about side effects of vaccine	3.9 ± 2.1	**0.873**	-0.004	0.148	0.048	0.205
Unconvinced of efficacy of vaccine	3.8 ± 1.9	**0.884**	-0.020	0.102	-0.023	0.142
Behaviour of someone I respect (role model)	1.7 ± 1.0	0.236	0.213	**0.783**	0.126	0.094
Have not received letter of invitation to be vaccinated against HBV	3.5 ± 2.2	-0.029	0.085	0.069	0.811	0.166
Insufficient information about the vaccine	2.9 ± 1.3	0.051	**0.871**	0.096	0.090	0.092
Insufficient information about the disease	2.6 ± 1.4	0.060	**0.902**	0.116	0.164	-0.104
Unable to afford the vaccine	2.4 ± 1.5	-0.001	**0.806**	0.113	0.208	0.168
Too busy/never enough time	3.9 ± 1.9	-0.095	0.187	0.055	**0.699**	0.309
Difficulty in obtaining the vaccine	2.5 ± 1.7	0.124	0.329	0.195	**0.742**	0.222
Fear of needles/injections	2.0 ± 1.5	0.366	0.063	0.017	**0.797**	-0.080
Not at increased risk	4.0 ± 2.2	0.320	0.041	-0.109	0.103	**0.587**
Someone’s (friend, partner, colleague) recommendation	1.4 ± 0.9	0.098	-0.069	**0.831**	0.069	-0.091
Physician’s recommendation	1.7 ± 1.1	0.076	0.235	**0.853**	0.025	0.001

Bold values indicate the highest loading weights.

**Table 5:** Exploratory factor analysis of the reasons for receiving the hepatitis B vaccine, questionnaire completed by healthcare workers at the Clinical Centre of Serbia, December 2015 (n=352)

Reasons	Mean score (n = 233)	Factor 1 Social influence	Factor 2 Knowledge of disease	Factor 3 Threat of disease
Recommendation of friend	3.2 ± 2.2	**0.770**	0.156	0.156
Recommendation of spouse/partner	2.4 ± 1.3	**0.836**	0.153	0.228
Recommendation of superior/supervisor	4.3 ± 2.2	**0.761**	0.237	0.153
Behaviour of someone I respect (role model)	3.6 ± 2.4	**0.821**	0.209	0.104
Recommendation of physician	2.7 ± 1.8	**0.636**	0.383	0.255
I provide care for hepatitis patients	4.9 ± 2.3	0.431	0.381	**0.549**
Previous needlestick/sharps injury	5.3 ± 2.6	0.109	-0.079	**0.815**
Possible restriction from patient care if infected	4.2 ± 2.1	0.229	0.448	**0.615**
Concern about professional liability	3.7 ± 1.7	0.074	0.436	**0.656**
Friend/co-worker developed occupational hepatitis	4.4 ± 2.3	0.295	0.425	**0.564**
Information letter from employer	4.0 ± 1.9	0.397	**0.698**	0.041
Information obtained from professional sources	5.4 ± 1.8	0.158	**0.790**	0.087
Information obtained from general media	4.0 ± 2.1	0.468	**0.597**	0.033

Bold values indicate the highest loading weights.

The model of potential reasons for not receiving the hepatitis B vaccine revealed five factors with an eigenvalue greater than 1, explaining 73.9% of cumulative variance (Table 6).

**Table 6:** Percentages of the variance explained of the vaccination-refusal and vaccination-acceptance related factors, questionnaire completed by healthcare workers at the Clinical Centre of Serbia, December 2015 (n=352)

**Vaccination refusal**		
**Factors**	**Eigen value**	**Percentage of variance explained**
Threat of disease	4.563	30.417
Knowledge of disease	2.464	16.428
Social influence	1.760	11.736
Access to care	1.267	8.447
Risk denial	1.033	6.885
**Total percentage of the variance explained**	** 73.913**	
**Vaccination acceptance**		
**Factors**	**Eigen value**	**Percentage of variance explained**
Social influence	6.178	47.521
Knowledge of disease	1.346	10.352
Threat of disease	1.027	7.897
**Total percentage of the variance explained**	** 65.770**	

Items clustered under ‘threat of disease’ explained the highest proportion of variance (30.4%) among those declining the vaccination, followed by ‘knowledge of disease’ and ‘social influence’ domains, explaining 16.4% and 11.7% of variance, respectively. Two other factors derived from this exploratory factor analysis model labelled as ‘access to care’ and ‘risk denial’ explained an additional 8.4% and 6.9% of variance of the reasons for not receiving the hepatitis B vaccine. The highest ranked reasons for hepatitis B vaccine refusal included ‘not at increased risk’ (4.0 ± 2.2), ‘concern about side-effects of vaccine’ (3.9 ± 2.1), ‘too busy/never enough time’ (3.9 ± 1.9) and ‘unconvinced of efficacy of vaccine’ (3.8 ± 1.9) (Table 3).

The exploratory factor analysis of the reasons for receiving the hepatitis B vaccine yielded three factors that explained 65.8% of variance among those accepting the vaccination (Table 5). Items clustered under ‘social influence’ had the highest contribution (47.5%) to explaining variance among the vaccinated sub-cohort. Two other factors in this model, ‘knowledge of disease’ and ‘threat of disease’ explained an additional 10.3% and 7.9% of variance, respectively (Table 5). The most highly ranked reasons for vaccination acceptance included ‘information obtained from professional sources’ (5.4 ± 1.8), ‘previous needlestick/sharps injury’ (5.3 ± 2.6), ‘provide care for hepatitis patients’ (4.9 ± 2.3), and ‘friend/coworker developed occupational hepatitis’ (4.4 ± 2.3) (Table 4).

Hepatitis B-related knowledge in our cohort of HCWs was assessed through 30 questions. The mean score in this questionnaire was 22.9 ± 4.8 (range: 8 to 30). The items and the proportion of correct answers are shown in Table 1.

The predictors of hepatitis B vaccination status among HCWs that were identified using logistic regression models are illustrated in Table 7.

**Table 7:** Logistic regression models of predictors of hepatitis B vaccination status, questionnaire completed by healthcare workers at the Clinical Centre of Serbia, December 2015 (n=352)

	Unadjusted models			Adjusted model		
	**OR**	**95% CI**	**p**	**OR**	**95% CI**	**p**
Age (years)	1.05	1.02 – 1.07	**< 0.001**	1.00	0.96–1.04	0.989
**Sex**	Reference category					
Female						
Male	1.54	0.90–2.65	0.113			
**Marital status** Married/cohabiting vs others						
	1.15	0.73–1.81	0.540			
**Occupation** Administrative staff, sanitary workers and others^a^ Physicians Nurses, medical and laboratory technologists	Reference category			Reference category		
	9.78	4.14–23.13	**< 0.001**	3.41	1.16–10.07	**0.026**
	5.27	2.48–11.17	**< 0.001**	2.52	0.93–6.84	0.068
**Work site** Inpatient wards Operating theatre Accident and emergency, haemodialysis unit Specialty ward/Intensive care unit Laboratory	Reference category			Reference category		
	2.45	1.41–4.23	**0.001**	1.43	0.73–2.79	0.293
	1.19	0.57–2.47	0.641			
	2.45	1.05–5.70	**0.038**	2.16	0.82–5.71	0.121
	1.45	0.26–8.13	0.675			
**Duration of work experience** (years)	0.95	0.93 – 0.97	**< 0.001**	0.95	0.92 – 0.99	**0.011**
**Blood exposure in the last year** None 1–5 times 6–10 times More than 10 times	Reference category			Reference category		
	2.39	1.24–4.61	**0.009**	1.78	0.78–4.05	0.171
	5.05	2.13–11.97	**< 0.001**	3.67	1.30–10.40	**0.014**
	2.88	1.53–5.43	**0.001**	1.95	0.86–4.41	0.108
**Sharps injuries in the last year** None Once Twice More than twice	Reference category					
	0.76	0.44–1.29	0.308			
	1.88	0.88–4.02	0.103			
	0.67	0.32–1.38	0.275			
Influenza vaccinations Never Once More than once	Reference category			Reference category		
	1.56	0.55–4.44	0.406			
	2.78	1.04–7.49	**0.042**	2.74	0.93–8.07	0.067
Seat belt use frequency Never Frequent Always	Reference category			Reference category		
	6.87	1.89–25.05	**0.003**	8.14	1.69–39.04	**0.009**
	5.03	1.53–14.46	**0.008**	4.79	1.15–19.94	**0.031**
Total hepatitis B-related knowledge score	1.15	1.09–1.22	**< 0.001**	1.10	1.03–1.17	**0.008**

CI: confidence interval; OR: odds ratio.

Bold values indicate statistical significance.

^a^ ’Others’ includes administrative staff, research scientists, sanitary workers, housekeeping, etc.

The unadjusted models revealed that significant predictive value for vaccination acceptance had the following variables: age, occupation, work site, duration of work experience, blood exposure in the last year, influenza vaccination, seat belt use frequency and total hepatitis B-related knowledge score. Furthermore, after testing for variables interaction and controlling the effect of potential confounders, the multivariate adjusted model has demonstrated that independent predictive value of hepatitis B vaccination status among HCWs remained significant for occupation, duration of work experience, blood exposure in the last year, seat belt use frequency and total hepatitis B-related knowledge score. Namely, this analysis showed that physicians had a more than three times greater likelihood of being vaccinated against hepatitis B compared with the occupational group consisting of administrative staff, sanitary workers and others (odds ratio (OR) = 3.41, p = 0.026). Additionally, this predictive model also demonstrated that with each year of work experience, the likelihood for vaccination acceptance declined by ca 5% (OR = 0.95, p = 0.011). Furthermore, participants who experienced unprotected blood exposure between six and 10 times in the last year had an almost four times greater likelihood of being vaccinated compared with those who did not report any accident in the previous year (OR = 3.67, p = 0.014). The HCWs who reported using seat belts frequently or always had an eight and five (respectively) times greater likelihood of hepatitis B vaccination acceptance compared with those who reported never using seat belts (OR = 8.14, p = 0.009; OR = 4.79, p = 0.031, respectively). Finally, after controlling for all of these potential confounders, the total hepatitis B-related knowledge score showed independent prognostic value in determining the HCWs vaccination status. Namely, adjusted logistic regression model revealed that with each one-unit increase in knowledge score, the likelihood of hepatitis B vaccination acceptance increased by 10% (OR = 1.10, p = 0.008).

## Discussion

Vaccination against HBV should be a moral imperative and responsibility for every health professional. Namely, successfully immunised HCWs not only protect themselves, but also prevent the spread of infection to patients and colleagues, and thus deliver safe healthcare. Despite over three decades of accumulated knowledge regarding the effectiveness and safety of hepatitis B vaccine, there is still a sizeable proportion of HCWs at a global level who never get vaccinated for various reasons.

According to the World Health Organization estimates, HBV vaccination coverage among HCWs shows remarkable discrepancy worldwide [2]. Namely, the lowest rates of hepatitis B vaccination acceptance were registered in countries such as Uganda (5%), Georgia (12%) [16], Kenya (13%) [2], Egypt (16%) [2], and Nigeria (18%) [2], while the highest rates were observed in the most developed countries, where typically, three quarters of HCWs are vaccinated against HBV [17]. It is clear that even in highly developed countries such as Sweden [18] the hepatitis B vaccination coverage is not satisfactory, and there is plenty of room for action, tailored for improving compliance with this vaccine. Given the acceptance rate among the HCWs in our study, the results highlighted the fact that almost half of the HCWs had not completed the course of vaccination, and 33.8% remained completely unvaccinated. These data confirmed the need for additional efforts to improve hepatitis B vaccine promotion and implementation in our healthcare community. As a matter of a fact, there is a lack of comprehensive organised efforts at this healthcare facility to ensure the maximum coverage among HCWs. Given that the hepatitis B vaccine in Republic of Serbia has been provided at no cost since 1989 and has been legally required for more than 25 years in this population group, employers have a duty to organise promotion, delivery and surveillance of HBV vaccination coverage. However, in our country, non-compliance with hepatitis B vaccination does not yet have any legal or professional repercussions. Therefore, in the authors’ opinion, to ensure optimum coverage there is an urgent need for surveillance boards to monitor compliance of hepatitis B vaccination acceptance among HCWs and subsequently consider charging penalties for non-responders. However, one of the first steps in creating an effective public health intervention is exploring factors responsible for vaccination acceptance as well as for refusal. Previous studies suggested that concern about side effects of the vaccine and its effectiveness, as well as the low perception of individual risk for HBV infection, were the major reasons behind poor compliance with hepatitis B vaccination [13,19]. Our results supported these findings in the literature, highlighting the importance of self-perceived hazard. This disturbing finding indicated a big knowledge gap that should be bridged as soon as possible.

On the other hand, when we consider potential reasons for receiving the hepatitis B vaccine, we observed that factors related to ‘social influence’ played the most important role in decision-making behaviour. In addition, results from other studies revealed that recommendations of a superior/supervisor, spouse or friend strongly influenced HCWs’ positive attitude towards hepatitis B vaccination [13].

In an attempt to elucidate independent predictors of hepatitis B vaccination status, physicians in our HCWs sample had more than three times greater likelihood of being vaccinated against HBV compared with the occupational group consisting of administrative staff, sanitary workers and others. This finding could be explained by the physicians’ greater educational and awareness status about hepatitis B and importance of its prevention, which is also supported by other authors [10,13,19]. Furthermore, duration of work experience also predicted acceptance of hepatitis B vaccination in our sample. According to these results, those with less work experience were more likely to be vaccinated. Possible explanation for this inverse association between shorter work experience and higher vaccination rate could be a result of greater acceptability of hepatitis B vaccine among younger HCWs due to more intensive educational programmes on HBV prevention during undergraduate medical studies, as commonly observed in other surveys [13,19-21].

The most common route of transmission of HBV in healthcare settings is needlestick injuries, especially those involving hollow needles. Approximately 70% of HCWs have reported needlestick injuries, with an average of two needle punctures per year. However, only ca 10–30% of needlestick injuries are reported to the authorities [22]. It is therefore reasonable that the frequency of this type of occupational accident has been recognised as one of the most prominent predictors of hepatitis B vaccination acceptance [13,19]. Our investigation also confirmed that participants who experienced unprotected blood exposure 6–10 times in previous year had an almost four times greater likelihood of being vaccinated compared with those who did not report any accident in the previous year.

Finally, after controlling for general acceptance of other preventive measures (frequency of influenza vaccination and seat belt use), in the last two steps of the multivariate analysis, the total hepatitis B-related knowledge score showed independent prognostic value in exploring the vaccination status in our sample of HCWs. Namely, adjusted logistic regression model revealed that with each one-unit increase in knowledge score, the likelihood of hepatitis B vaccination acceptance increased by 10%. The results from studies in various setting also indicated that greater knowledge of both HBV infection and vaccination resulted in positive attitudes among healthcare providers, and sustained their beliefs in the safety and efficacy of the vaccine [13,19,23,24]. This finding suggests that education aimed at improving HCWs’ HBV-related knowledge is likely to be a crucial component in increasing hepatitis B vaccination acceptance. It has been suggested that repeated educational programmes may be the most effective way to achieve this goal [13]. Therefore, under present conditions, it is the responsibility of non-vaccinated HCWs themselves to be aware of their hepatitis B infection risk and the importance of primary prevention, and we suggest healthcare facilities in Serbia should be required to establish HBV vaccination as a prerequisite for employment.

Some limitations of the present study need to be kept in mind when interpreting the results. Firstly, this investigation was performed at one national clinical centre, and thus selection bias cannot be excluded. Secondly, cross-sectional design captures association but does not allow for determination of causality or temporal sequence. Thirdly, an information bias should be acknowledged, because this study relies on self-reported data, which may be subject to over- or underestimation, potentially distorting results. Another drawback of this study is the anonymous nature of data collection, as we were not able to track subjects who were not vaccinated and offer them HBV vaccine. Despite limitations, there are several advantages to our study, including the fact that such a study was conducted for the first time in Serbia. We recruited a representative sample of the HCWs from a large referral healthcare facility. Because of this, we hypothesise that the results of our study could be generalised to the total HCW population of the country.

In conclusion, the findings of our study showed that a knowledge gap exists around Serbian HCWs’ awareness of hepatitis B vaccination, leading to suboptimal coverage. Further vaccination implementation efforts should emphasise the comprehensive involvement of HCWs in continuing education about occupational risk, liability, safety and effectiveness of hepatitis B vaccination. Therefore, there is a need for clear, well-planned national policies and guidelines, including the possibility of mandatory HBV immunisation within the Serbian healthcare environment.
